# Hospital Reform Conference

**Published:** 1899-10-14

**Authors:** 


### The Institutional Workshop.

### HOSPITAL REFORM CONFERENCE.

### THE INQUIRY SYSTEM—PAYMENT BY PATIENTS—PROVIDENT DISPENSARIES.

Under the auspices of the Hospital Reform Association, a conference was held at St. Martin's Town Hall on the 10th and 11th inst. There were three sittings—two on the former date, in the afternoon and evening respectively, and one on the 11th. It had been intended that each of the three subjects for discussion—viz., "The Inquiry System," "Payment by Patients," and "Provident Dispensaries"—should occupy one sitting, but in consequence of so many papers being submitted to the conference relative to the inquiry system, the consideration of that subject somewhat overlapped the evening proceedings, a course that met with some little disapproval among those then assembled. The attendance was not large, but considerable interest was manifested in the proceedings by those present.

#### Tuesday Afternoon.

The sitting on Tuesday afternoon was presided over by Dr. W. Knowsley Sibley, of the North-West London Hospital.

In opening the proceedings, the Chairman intimated that four papers were to be read on the subject of the inquiry system. The Hospital Reform Association, he said, was founded in 1896, at which time the question of hospital abuse was beginning to attract public notice, being then in the hands of a few specialists. The main bulk of the hospital authorities, however, were so far in ignorance of the question that they showed righteous indignation at the very word "abuse." In London, especially, they said such a thing did not exist, and as to there being any necessity for inquiry—well, the whole thing was more or less pooh-poohed. The first move made was when the Hospital Sunday Fund in 1897 passed a resolution that in the matter of the distribution of their funds they were going to give special merit to those who had an inquiry officer. As this was a body which could touch the purses of the hospitals, they listened to it and more or less investigated the alleged abuses, instituting in the end some kind of inquiry. Many of the officers who had been appointed were rather inefficient, but one or two had an inquiry officer of the right kind. He was sorry to see that the Prince of Wales's Fund, which had done so much good work in various directions—and which had recently instituted a special visiting committee to report on the efficiency or otherwise of the administration—was at present rather adverse to anything of the kind. They said their work was to go into statistics, &c., and they had nothing to do with the question of who was treated or how they were treated—that rested with the individual hospitals. He regarded that as a mistake, feeling as he did that the Fund would become more popular if it would follow more or less in the lines of the Hospital Sunday Fund, and give merit to those hospitals where a proper inquiry took place.

Mr. Douglas Dent, of Bristol, in the course of a lengthy paper upon the Inquiry System, said (1) the main thought that had prompted the charity of those who gave money every year to hospitals was that the suffering and deserving poor might not go uncared for. They were all agreed that free treatment such as the author of the paper defined should be accorded to the poor, and they were equally sure that if treatment of the same character ought to be given to other than the poor it ought not to be free, but ought to be paid for honestly, according to the means and resources of the patients. Here charity should not dare to interfere; the patient should spurn the interference if offered. Alas! however, the temptation had been too great for the patient, while charity had been too supine to object, too selfish refuse. The natural consequence was an alarming increase in the number of patients, which proceeded in a quickening ratio as years passed, threatening to end in the bulk of the population demanding free treatment, the managers of our hospitals using the fact of the increase in demand as a lever for obtaining more money from the charitable, and long-suffering charity, at last awakened, cutting off supplies and rate-supported institutions taking the place of what it ought to be the pride of Christian love to maintain in utmost efficiency for the really poor of the land. As regards London, statistics had been strikingly stated by Sir Henry Burdett in his letter to the Press of August 22nd, 1896, comparing the growth of hospital patients and the population of London. Sir Henry said, "Comparing the figures for 1893 with those for 1873 we find that whereas in 20 years the population has increased by about 28 per cent. the number of patients relieved by the hospitals has increased by a little over 75 per cent." The same thing was going on in the provinces. (2) To those who had thought about all this it seemed almost impertinent to name so simple a course—in order to check such misuse of our medical charities as existed—as the word "inquiry" suggested. Quoting again from Sir Henry Burdett's letter, "Once enact that whilst every hospital is free to the deserving poor no patient will be permanently retained in a free bed unless he can satisfy the authorities that he is entitled by his circumstances to the free medical relief, and hospital abuse would cease altogether and for ever so long as this enactment was intelligently enforced throughout all departments of every voluntary hospital." All authorities required to be satisfied that the objects for which they existed were not frustrated by failure of compliance with rules for their attainment. Hospital authorities, however, were satisfied as a rule by the mere application for free treatment convincing them that the applicant was entitled to it. Lately some of them were more strict, but how few went to the wicked extent of testing the truth of the answers they received. All were really agreed that inquiry should be made, though not agreed as to the extent to which it should be carried. Who would say that in the case of voluntary hospitals the authorities were not entitled to be satisfied that the applicant was entitled by the circumstances to free medical relief? And yet how difficult it was to get the managing committees to recognise their responsibilities to the charitable public, or to get the latter to insist on the committees doing what was their plain duty. (3) The inquiry was said to be so difficult, so impossible, even. Was this so? Until eleven years ago he really had never heard of any method that made inquiry possible. But he had learned that it was possible to make an inquisition safely, easily, and completely into the truth of any statement that was desired to be tested. This inquisition might cover the whole history of an applicant, or be limited to part or parts of it. For the purpose of deciding whether an applicant was entitled to free medical treatment, the inquiry could be limited strictly according to the conditions under which such treatment was offered, although, as he thought, it should be extended to include all that was needed in order effectively to relieve the applicant and make him independent of charity, if necessary and if possible. The other side of the question, from the point of view of the applicant, must be considered also. If the inquiry were not the difficult matter it was once thought to be, was it hard on the applicant? Some said it was hard, and even unjust to the poor. Here, again, experience must be relied upon for an answer to the charge of hardness, and he maintained that such inquiry was not resented by the honest applicant, who deserved all sympathy and consideration at their hands; that, on the contrary, it was, as a rule, welcomed, and its reasonableness was fully allowed. Why, indeed, should it be otherwise? On the other hand, if the attempting deceiver should resent this inquiry, who should or would pity him? Yet even here his (the author's) own experience was that the deceiver seldom vented his displeasure, if he felt it, and could be brought to acknowledge the justice of the operation, always provided it was conducted in a proper, kindly manner. Anyway, what better plan could there be for securing that imposition was not practised? Hospital authorities had had no hesitation in laying down very strict regulations in other directions, nor in keeping out-patients waiting an inordinate length of time, and he thought it would be admitted by all reasonable people that, if clear regulations in regard to inquiry were published and adhered to, no complaint would be heard, at least, from those for whom the charity was established and, in theory, was maintained, and who had the only right to complain. If these and others could only be convinced that inquiry was not difficult, but comparatively easy, and not hard on the poor, but just to them, the battle in a very hot part of the field was won. The positions he had tried to induce his hearers to occupy were: (1) That it was time to restrict the benefits of charitable medical relief to those for whom alone they were intended, namely, the suffering deserving poor, and so check their misuse by those otherwise provided for, or able to pay; (2) that inquiry into the fulfilment by applicants of the conditions under which they were entitled to such relief was the true remedy for, and preventative of such abuses; (3) that this inquiry was not a difficult, but a comparatively easy operation, and involved neither hardship nor injustice to the applicants; (4) that the result of the adoption of the principle would be for the good of the poor, the charitable public, the medical profession, and the hospitals.

Sir William Broadbent characterised the paper just read as the best exposition of the principles upon which charitable relief ought to be conducted he had ever heard. There was no single word in it from which he dissented. As a hospital physician to many out-patients and in-patients he had seen from time to time that the services of the doctors were given to cases which were not really proper cases for charitable assistance. But the doctors themselves felt perfectly hopeless. It was not for them, when such a case came before them, to take upon themselves the duty of protesting and inquiring. It was through the agency of the Charity Organisation Society, through the methods which it had adopted and which were not only efficient and trustworthy, but in essence kindly and just—it was through the work of that society that he had come to entertain some hope that hospitals might be relieved from work which was not their own, that the malversation of charitable funds—for that was what it amounted to—might be prevented, and that the suffering poor might come by their rights. That was what they had in view, and that such a thing was possible he had been at any rate hopeful; indeed, having heard the paper just read, he was confident that this end could be accomplished. The inquiry instituted by many of the hospitals was honest enough, was in a certain degree efficacious, and was to some extent deterrent, but it could not be the complete inquiry which alone made it possible to deal with the abuses which more or less prevailed. He could relate instances of abuse almost as startling as that related by the chairman. He did not say such cases were numerous, but the fact that they were possible almost amounted to a scandal, and while cases of that kind were exceptional, other cases where people well able to pay and who would be better in themselves for paying, but who did not, were much more numerous. As to inquiries made at certain hospitals, unless they were made at all hospitals and upon a uniform principle, all they would do would be to shift unsuitable cases, and unfortunately there were institutions which were only too ready to accept these unsuitable cases, totally disregarding the interests either of charity or of the patients themselves or of the medical profession. It was only when the inquiry could be complete—and he was glad to hear from the paper that that was much more possible than was supposed—that they would arrive at a solution of the question.

Colonel Montefiore next read a paper on the same subject. He thought it would be conceded without hesitation that our charitable hospitals were instituted by the benevolent to give medical or surgical advice and good nursing to those people who were unable to afford them in their own homes. For the most part the patients in the wards of these institutions were those who could not obtain such necessities for themselves. But in the out-patient and casualty departments many patients were admitted whose complaints could be far better treated in their own homes by the local general medical practitioners. There were patients continuously treated in these departments whose social and pecuniary position was such that the advice and medicine given to them were worse than useless, and whose physical condition was not serious nor acute enough to warrant their admission to the wards. These patients required food, shelter, firing, and other necessaries, as well as medicine, and should be referred to those to whom authority had been given to deal with such cases. He therefore believed that no hospital board or committee of management was doing its duty to the charitable public who gave it money nor to the patients who flocked in such large numbers to its outdoor department if the persons were not classified and examined with a view to ascertaining (1) those whose complaints were of such a nature as to require the attention of expert consultants, and those who could be equally well treated outside the hospital; (2) those able to pay the ordinary fees of the general medical practitioners in their own locality; (3) those able to make small periodic payments while in health to provide themselves and their families with medical attendance when ill; and (4) those who required charitable assistance besides medical or surgical advice and medicine. He believed investigation into the social circumstances of the out-patients should be made, not only with a view to check abuse, but with a view to raise the applicants, so that after full care had been bestowed upon them they should become independent of the charity in similar circumstances. To ascertain how to so raise the applicant it was necessary to investigate thoroughly, and to do this it was imperative to throw much of the work on charitable agencies outside the hospital. It was impossible to discriminate by asking a few set questions, and the selection of seemingly unfit cases by a person who, although zealous, was untrained in charitable work, was a mere waste of time. The inquiries must be made in conjunction with the medical officers, and therefore such a plan of inquiry as would work best at all hospitals was that now carried on at the Royal Free Hospital, where an almoner was employed who had been trained and had had considerable experience.

Mr. G. A. Hawkins-Ambler, F.R.C.S.E., Liverpool, question was largely one of education and enlightenment. He did not know what the public wanted, and did not think it knew itself, but the drivel they read about hospitals being the only source of intelligent or suitable treatment for the poor, and of specialists as being the only proper persons to be entrusted with the care of wealthier patients, was extraordinary. The source and intention of subscriptions formed not the least important part of the question of hospital abuse, and called for the gravest consideration. Large firms gave apparently handsome subscriptions, but in return extracted from the hospitals more than their share of attention for workmen; just as the wealthy classes sent their servants for the treatment to which their guinea did not entitle them. In further remarks Mr. Hawkins-Ambler said the infamous imposition of the public on all branches of the medical profession, which was also an imposition on charity and a sequestration of the resources of the needy poor, was a crying abuse. No words were too strong to denounce it, no efforts could be better spent than on abating it. The charitable were, fortunately, discovering that their sacrifices were being made in many cases for the benefit of persons better able to pay for assistance than the subscribers themselves, and there could be only one result, viz., the stoppage of the source of that beneficent help that tided the suffering through times of stress—carrying them safely over the gulf of pauperism that ever yawned in front of the poor, be they disabled ever so temporarily—and an increase in the bitterness of class feeling. The vitality of hospital abuse lay in the fact that there was no public opinion against it. It was here, and it was candidly and quietly accepted. The public needed, above all, to be educated to a sense of responsibility for themselves, their families, and their social obligations, of the need of deprivation of those eternal amusements and luxuries without which modern society could not get on one day. How were they to be educated? He thought the inquiry system the best means of all. Let them decide on a wage limit; let it be fair, let it be capable of expansion for difficult cases, for special cases, for prolonged treatment, for costly treatment, for all the considerations that sickness brought in its train, but set one and keep to it pretty strictly. Each hospital should have its inquiry officer, who should be the centre of the institution around which it did its work.

Mr. Arnold W. W. Lea, M.D., B.S., F.R.C.S., Dr. Lorimer Hart, and others addressed the meeting.

Dr. Isambard Owen was inclined to think that the more the London hospitals would unite with each other the better the inquiry system would work. If every hospital were going to establish its own staff of detectives and its own agents in all parts of London the process would inevitably become ruinously expensive, but if the inquiry for all hospitals in London could be made by one association—whether the Charity Organisation Society or some new establishment—it was quite possible, with efficient agents in every part of London, that adequate inquiries might be made. They heard a great deal about abuse in the out-patient department of the hospitals, but the greater part of hospital abuse seemed to lie in the casualty practice. The evil of the present system, as regarded the medical profession, had been a little too much looked at as though it consisted simply in giving gratuitous advice and drugs to a certain number of persons who might otherwise have paid a small fee to a medical man; but that seemed to him to be the least part of the evil as regards the medical profession. The great evil was the inadequate rate of remuneration paid, especially to those in general practice. The rate had gone down by some 50 per cent. in the course of the last few years, and a large element in the reduction of that rate must be afforded by the enormous casualty, as well as out-patient practice done by the general hospitals. The rate of remuneration must necessarily go down when an ordinary man had to compete with an institution which was ready to offer a bottle of medicine to all comers without any payment. That evil was, to a certain extent, stopped by the practice of limitation which prevailed, for instance, at St. George's Hospital, where every patient who came was looked upon as a possible case for admission as in-patient, and no patient was treated as casualty unless it was a case of accident or some sudden seizure in which the postponement of treatment for twenty-four hours would be attended with serious results.

Mr. Thomas Bevan (vice-chairman of the Distribution Committee of the Hospital Saturday Fund) said the commitee of that fund were at one with this association in trying to settle this vexed question.

The conference then adjourned.

### Tuesday Evening.

The Rev. A. G. Russell-Wakefield presided over the evening meeting, and said that although the meeting was primarily arranged for the consideration of the question of "Payment by Patients," it had been decided to allow discussion upon the subject which had been under consideration during the afternoon. It would, he went on to say, be impossible for any clergyman working in London not to be aware of the necessity for something being done as to payment by patients. They had to distribute an immense number of letters, and they knew that sometimes there were abuses in consequence of the present system, notwithstanding that they did their very utmost to prevent such abuse. On the other hand, there were many people who hesitated to take a letter simply because it was absolutely free; they felt it would be more fitting that they should pay something. If payments were made customary, he thought that there would be less abuse. He was constantly asked for free letters by people who could well afford to pay a considerable sum to their own private doctors, and he was asked for letters by people on behalf of dependents in many cases where the applicants would do a great deal better to themselves provide a doctor for that dependent. Most bare faced applications were made at times, and it was perfectly abominable that such should be the case. There was also another side to the question, and that was that it was a cruel thing that doctors, living in poor districts, who were always ready, in season and out of season, to be at the beck and call of the poor, and who had no opportunity of making anything more than the barest competency, should have taken away from them those who ought, and, but for the hospital letter, would go to them and pay a fee. The labourer was worthy of his hire, and as the doctors themselves would be the last people to urge this, he, as a parson, ventured to do so for them. It would be better, moreover, for the self-respect of the people—in fact, better in every way—if some little payment were made.

The Hon. Secretary (Dr. T. Garrett Horder) read a paper contributed by Captain Acland, the chairman of the committee of the Dorset County Hospital, at Dorchester, in which the writer described the system in force at that institution.

Mr. C. S. Loch (secretary of the Charity Organisation Society) said that, if the facts put forward by Dr. Waring were true, a *prima facie* case was made out as against those who approved of the present system as now conducted. To examine such a case, however, it was necessary that there should be verification. They had already had several inquiries, but without any real verification; the results of the inquiries were absolutely nugatory. It should be made known whether those who wished to carry on the system of hospital administration on the present lines were willing to have a thorough inquiry, and the general practitioner, who was one of those concerned, should be a party to that inquiry. There was still a further difficulty. There was a most unfortunate dissidence between medical men in the hospital and medical men outside. It was not very real, but in a manner it existed. If hospitals desired to introduce the pay system, which was said to be detrimental to the profession, what was the position of a medical man on the staff of the hospital? He must either accept what the committee said or go. If that were a fair statement there was room for inquiry, and that by medical men. The independence of medical men on the staff of hospitals should be preserved from a professional standpoint. If the hospital system was underselling the general practitioner, as was suggested, the latter should be very careful to collect evidence on the subject. The present position of hospitals meant that they would have to introduce a system by which payment would become a distinct part of the yearly assets of a hospital, or, on the other hand, municipalisation would be advocated to a larger and larger extent. The speaker contended that the onus of responsibility lay chiefly with the medical men themselves if a solution of the present difficulties were to be found.

The Hon. Sydney Holland (Chairman of the London Hospital) was asked to speak, but said that it was hardly fair to ask him to reply in five minutes (the time limit laid down) to arguments contained in a paper the reading of which had occupied fully twenty minutes.

Mr. Dennis Vinrace was in opposition to anything like payment, feeling as he did that there should be absolute and definite demarcation between payment and gratuity. It was very clear that abuses existed, and every practitioner could give ample evidence of such abuse. In addition to his objection to payment, the speaker deprecated the system of letters, which indicated a *quid pro quo*, and which were often distributed in a most improper and indiscreet manner. He contended it was idle to suggest that the medical profession benefited to a vast extent by the crowds of out-patients, and that this counterbalanced the benefit which accrued to the public weal. He held that it was absolutely demoralising to give and receive treatment in this way.

The Chairman, touching upon the question of underselling, suggested that it would hardly be possible for the hospitals to undersell some medical men, whose limit in the way of payment was very low indeed.

The Hon. Secretary appealed to Mr. Sydney Holland, who, he said, had been the chief means of introducing patients' payments in the out-patient department of the London Hospital, to make a few remarks on the question.

Mr. Holland said he objected to this Hospital Reform Association, which was got up by a lot of gentlemen living in Wales.

The Hon. Secretary demanded a withdrawal of that statement, remarking that it was not true, and that the Association was composed of gentlemen living in all parts of the country. Mr. Sydney Holland should stick to the truth. He (the speaker) happened to live in Wales, but the members were spread all over the country.

Mr. Holland declined to withdraw.

The Secretary: Mr. Sydney Holland is nothing if not personal.

Mr. Sydney Holland said he had strong views upon the question under discussion, but he could not expound them in five minutes.

Mr. W. Grisewood (of Liverpool) considered the system of payments should be based upon what the patient was able to pay.

Mr. Bunn (secretary of the Hospital Saturday Fund) said Dr. Rogers stated that the hospitals making a charge did not publish the fact, and that it would be more honest if they were to do so. Now the two hospitals that received the largest sum from patients did let the public know; in fact, they made the payment by patients the basis of their appeal to the public for funds. He did not think the introduction of the small fee of 3d. in the out-patient department of the hospitals referred to had in any degree changed the class of the patients, and that was a most important point.

In reply to a question Mr. Sydney Holland said the London Hospital adopted the system in April, 1898, and Guy's adopted it six years ago.

Mr. Bunn (continuing) said that the people paid the money cheerfully, and instead of feeling they were purchasing medical relief, in hundreds and thousands of cases the money was given in gratitude for the benefits received.

Miss Beattie said it would be interesting to know the number of patients who had to go to the Charity Organisation Society before they could get the 3d. to take to the hospital.

Mr. Holland wished it to be distinctly understood that the charge made at the London Hospital was not for treatment, but merely for medicines and bandages. Where no medicines or bandages were supplied the money was given back.

A vote of thanks to the chairman brought the sitting to a close.

Wednesday Afternoon.

Mr. Timothy Holmes occupied the chair at the sitting on Wednesday. The subject of "Provident Dispensaries," he said, was one in which he had long been very much interested; in fact, ever since Sir Charles Trevelyan brought the matter prominently before the public he had been more or less at work on the Metropolitan Provident Medical Association. Everyone connected with practice in London knew the difficulties in the way of starting an association of that kind. The poor had long been accustomed not only to be provided for, from a medical point of view, gratuitously, but almost to be scrambled after by various institutions, and considering the prestige that hospitals always had over private practitioners, it was almost impossible to carry out such a scheme as the association referred to had propounded. However, thanks to the zeal and the energy of the people working the scheme, a commencement had been made which was very gratifying. There was a large number of provident dispensaries now in London which were self-supporting, and in which the medical officers received a remuneration which, at any rate, made it worth their while to continue their services. He hoped the great idea of his deceased friend would be successfully realised. If they could get the system set up, no doubt a great step would be taken towards the reformation of the hospital out-patient system.

Mr. Warren, secretary of the Metropolitan Provident Medical Association, read a paper descriptive of the objects and organisation of that institution, stating, *inter alia*, that with one exception, the most successful provident dispensaries were those situated farthest from hospitals. The Royal Free Hospital had set an example to the other hospitals in the matter of co-operation with provident dispensaries.

Discussion upon the paper was taken part in by Mr. A. Smith, Mr. Grisewood, and Mr. Bousfield, president of the Metropolitan Provident Medical Association, one of the principal subjects discussed being the attitude of medical men towards provident dispensaries.

A paper on "Reform in Medical Contract Work," by Dr. W. Richardson Rice, of the Public Medical Service, Coventry, and a communication from the medical staff of the Coventry Provident Dispensary, were also read.

